# Immunomodulatory role of RNA modifications in sex hormone-dependent cancers

**DOI:** 10.3389/fimmu.2025.1513037

**Published:** 2025-05-08

**Authors:** Yujia Jiang, Xiaolan Liang, Hongyi Sun, Ping Yin, Jing Zhou, Chaoqin Yu

**Affiliations:** ^1^ Gynecology Department of Traditional Chinese Medicine, The First Affiliated Hospital of Naval Medical University, Shanghai, China; ^2^ Department of Reproductive Medicine, Shuguang Hospital Affiliated to Shanghai University of Traditional Chinese Medicine, Shanghai, China

**Keywords:** RNA modification, sex hormone-dependent cancer, tumor microenvironment, tumor-immunology, epigenetics

## Abstract

Recent studies have identified that RNA epigenetic modifications, including m6A, m1A, m5C, etc, play pivotal roles in tumor progression. These modifications influence mRNA stability, RNA processing, translational efficiency, and decoding precision. However, comprehensive reviews detailing the connection between m6A RNA modifications and hormone-dependent cancers in both male and female populations remain scarce(breast cancer, ovarian cancer, and endometrial cancer, prostate cancer). In this article, we explore the cellular and molecular roles of various RNA modifications alongside the key elements of the tumor microenvironment. We examine how these RNA modifications influence the development of hormone-dependent cancers through their impact on immune mechanisms. By enhancing our understanding of the function of RNA modifications within the immune systems of four specific tumors, we offer fresh insights for their potential applications in diagnosis and treatment.

## 1 Introduction

Tumor development is influenced by multiple factors, among which a subset closely associated with hormones, known as hormone-dependent cancers, among the most typical ones are breast cancer, ovarian cancer, endometrial cancer, and prostate cancer (BC, OC, EC, and PC). Their development and treatment are intrinsically linked to hormones, mainly including progesterone receptor (PR), estrogen receptor (ER), androgen receptor (AR) and human epidermal growth factor receptor 2 (HER2). In women, BC is the first malignant tumor with the highest incidence rates and is also the most typical hormone-dependent intermediate utilized (1). In addition, complex molecular bidirectional interactions between hormone receptors (HRs), including ER, PR, and HER2 are present in BC (2, 3). Endocrine therapy (aromatase inhibitors and anti-estrogen therapy or anti-estrogen therapy alone) is the standardized method and is the backbone of adjuvant therapy that significantly reduces the risk of recurrence and mortality (4). Endometrial cancer, on the other hand, is the female malignant tumor with the fastest growing mortality rate, which tends to show elevated ERα levels and promote PR expression (1, 5, 6). Patients are positive for both ERα levels and PR expression tend to have well-differentiated tumors and may be responsive to hormone therapy, resulting in a relatively good prognosis (7). In addition, ovarian cancer is an essential branch of female malignant tumors, and postoperative hormone replacement therapy is necessary to achieve a better quality of living for patients (8). Similar to women, the most prevalent malignant tumor for men, prostate cancer, is significantly affected by androgens. Hence, the basis of prostate cancer treatment is anti-androgen therapy (9, 10). However, their mechanisms of occurrence remain unspecified, and treatment outcomes remain unsatisfactory. This review emphasizes the importance of discovering alternative and targetable molecular pathways that could provide novel therapeutic opportunities.

RNA modifications refer to chemical alterations of RNA nucleobases or ribose molecules. Presently, over 150 distinct modifications have been documented. Pseudouridine Ψ was discovered in the 1950s as the first recognized RNA modification (11). Among the most prevalent mRNA modifications, N6-methyladenosine (m6A) was identified in 2011 (12–14). Other RNA modifications, such as m1A and m5C, have been identified and extensively studied in recent decades (15, 16). RNA methylation has an impact on almost the entire mRNA life cycle - starting from mRNA transcription, mRNA splicing, specific structure, stability and subsequent translation and finally degradation (17–21). Although much of the research has focused on the role of m6A in hormone-dependent cancers, this review also examines the impact of other RNA modifications, such as m1A, m5C, m7G, mcm5s2U, A-to-I, and Ψ. These modifications can be investigated using emerging techniques like RNA immunoprecipitation (RIP), chromatin immunoprecipitation (ChIP), and single-cell omics (22–25).

The role of the immune system in cancer development has attracted increasing attention, particularly concerning the complex immune components within the tumor microenvironment and the adaptable mechanisms of immune evasion. Consequently, immunotherapy has emerged as a novel approach in cancer treatment, aimed at remodeling the immune system and reactivate anti-tumor immune responses to avoid tumor escape (26). Various immunotherapeutic strategies have shown substantial promise in treating a wide range of cancers, predominantly involving immune checkpoint inhibitors like PD1, PD-L1, and CTLA4, antibody-drug couplings, and cancer vaccines (27). However, fewer immunotherapeutic agents have been approved for clinical use in hormone-dependent cancers (28–31). As a distinct regulatory mechanism, RNA modifications, exemplified by m6A, has garnered increasing attention. Tumor-derived intrinsic signals and environmental stimuli can drive aberrant expression and activity-modifying regulators of many RNAs, leading to abnormal RNA modifications, which are essential for shaping the tumor microenvironment and immune escape (32, 33).

Although a substantial body of literature has accumulated on RNA modifications in cancer, the research focus has predominantly centered on m6A modification and its associated enzymatic machinery, with relatively limited exploration of other RNA modification types and a notable paucity of systematic reviews. While tumor immunology remains a prominent research frontier, investigations that integrate RNA modifications with tumor immunity to elucidate their epigenetic regulatory mechanisms remain relatively scarce. Notably, for hormone-dependent tumors, there is a conspicuous lack of comprehensive discussion regarding the potential shared immune regulatory mechanisms and epigenetic modification patterns that may arise from their similar endocrine microenvironment (34). This paper will focus on how RNA modifications play an immunomodulatory role in hormone-dependent cancers, including breast, ovarian, endometrial and prostate cancers (BC, OC, EC, and PC). The mechanisms and implications of prevalent RNA modifications will be explored. Specifically, we aim to elucidate the effects of RNA modifications in diverse immune cell types within hormone-dependent cancers.

## 2 RNA modification in sex hormone synthesis

### 2.1 Concepts of different RNA modifications

#### 2.1.1 m6A

**Figure 1:**
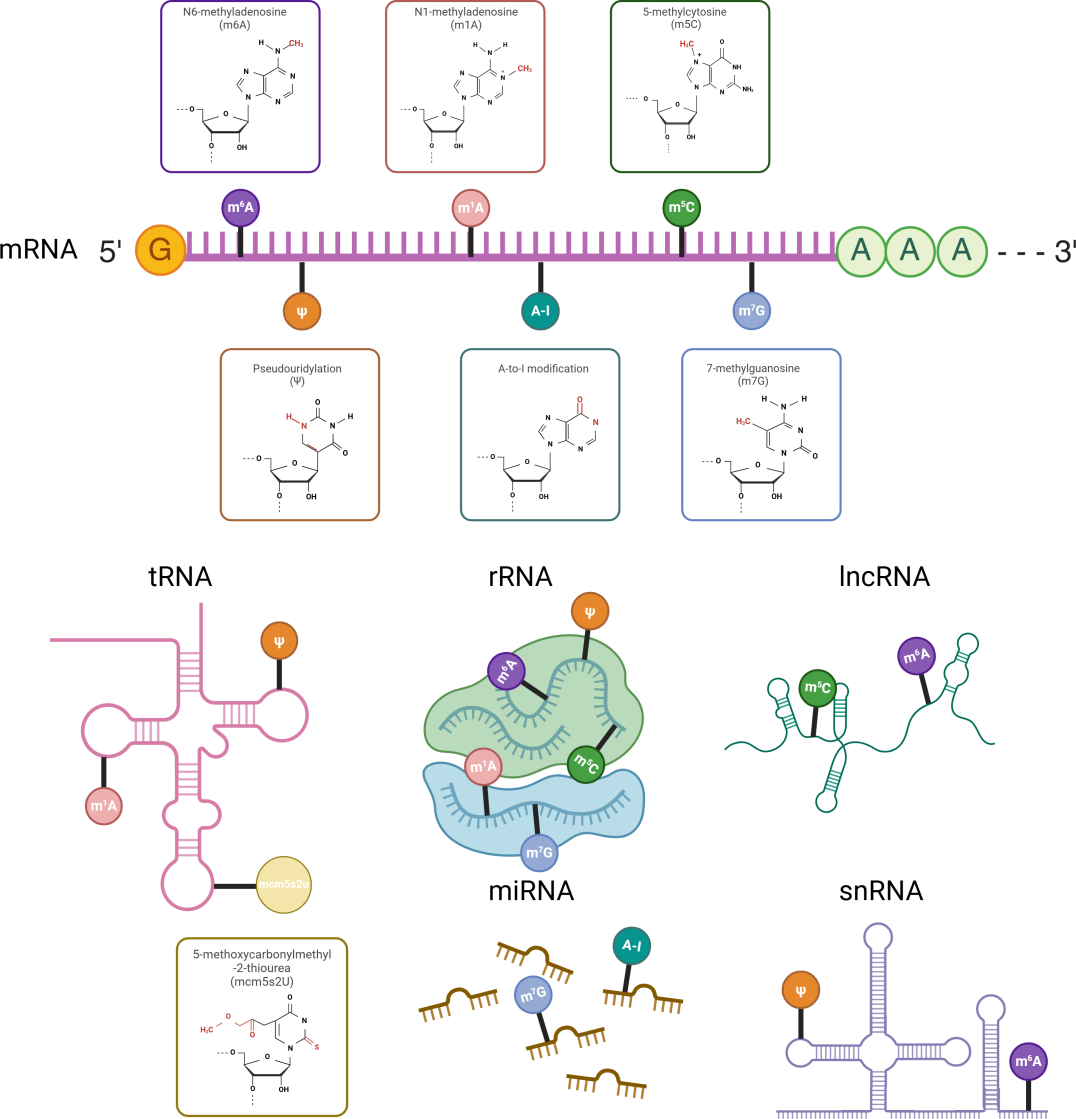
RNA modifications across different RNA types with chemical structures highlighted on the ribose moiety. Different RNA types undergo distinct chemical modifications that influence their stability, processing, and function. mRNA is primarily modified by m⁶A, m¹A, m⁵C, m⁷G, A-to-I, and Ψ, with m⁶A being the most prevalent. tRNA mainly carries m¹A, Ψ, and mcm⁵s²U, while rRNA undergoes m⁶A, m¹A, m⁵C, m⁷G, and Ψ modifications. lncRNA is modified by m⁶A and m⁵C, snRNA by m⁶A and Ψ, and miRNA by m⁷G and A-to-I. The chemical structures of these modifications are marked on the ribose moiety.

N6-methyladenosine (m6A) is defined as the methylation of adenine at the N6 position within RNA molecules. It represents the most prevalent modification in eukaryotic lncRNAs and mRNAs, and has also been detected in rRNAs, snRNAs, and tRNAs (**Figure 1**, 35). The m6A modification is conserved across yeast, mouse, and human mRNA, and is enriched in the RRACH (R = G or A; H = A, C, or U) consensus sequence (36–38). The m6A modification is primarily facilitated by the methyltransferase termed the “writer”, the demethylase known as the “eraser”, and recognition proteins referred to as “reader”. The writer assembles the m6A methyltransferase complex (MTC), which catalyzes site-specific methylation that can be reversed by the eraser. The reader proteins bind to methylated m6A sites and transmit downstream signals, thereby acting as post-transcriptional gene regulators. M6A writers mainly include METTL3, nephroblastoma 1-associated protein (WTAP), KIAA1429 (VIRMA), RBM15, METTL16, METTL14, HAKAI, and ZC3H13 (KIAA0853) (39). To date, only two types of erasers, FTO and ALKBH5, have been recognized (40). The readers involve the YTH family, the HNRNP family, and the IGF2BPs family (39). As an important component of epigenetics, m6A modification and these regulatory proteins are involved in various biological activities in which they play a regulatory role (40–43).

As the predominant “writer,” METTL3 serves as the core catalytic component of MTC, yet it remains inactive without METTL14. Although METTL14 lacks intrinsic methyltransferase activity due to the absence of an S-adenosylmethionine (SAM) binding domain, it aids in substrate RNA recognition and forms methyltransferase structural domains (MTDs) with METTL3 as a heterodimer (44). The MTD structural domains in isolation do not possess methyltransferase activity and necessitate the zinc-finger domain (ZFD) of METTL3 to become enzymatically active (45, 46). Wilms tumor 1-associating protein (WTAP), though not enzymatically active, assists in mRNA methylation by interacting with and recruiting the METTL3-METTL14 complex to target mRNA sites (47, 48). Further studies have identified VIRMA (49), ZC3H13 (50), RBM15/15B (51), and HAKAI (52) as additional cofactors of the METTL3-METTL14 complex. Besides METTL3, three distinct enzymes—METTL16, METTL5, and ZCCHC4—have been recognized as eukaryotic m6A methyltransferases, each responsible for incorporating m6A into U6 small nuclear RNAs (snRNAs) (53), 18S ribosomal RNAs (rRNAs) (54), and 28S rRNAs (55), respectively.

FTO and ALKBH5, known as m6A erasers, act as demethylases that catalyze the conversion of m6A to adenosine. FTO was the first m6A eraser identified, exhibiting specific oxidative demethylation activity against abundant m6A residues on RNA (13), while ALKBH5 was the second eraser discovered (40). FTO demethylates internal m6A residues on mRNAs and U6 RNA, as well as N6,2-O-dimethyladenosine (m6Am) on mRNAs and snRNAs, N1-methyladenosine (m1A) on tRNAs, 3-methylthymine (m3T) on single-stranded DNA (ssDNA), and 3-methyluracil (m3U) on single-stranded RNA (ssRNA) (56, 57). ALKBH5 localizes within nuclear speckles and aids in the assembly of mRNA processing factors, primarily acting on substrates like nuclear nascent RNAs (ssRNAs) (40).

The primary readers of m6A are the YTH family proteins, including YTHDF1, YTHDF2, YTHDF3, YTHDC1, and YTHDC2, which contain an m6A-binding pocket within their YTH structural domains (58).YTHDF2, the first to be identified, promotes the degradation of cytoplasmic targets by recruiting CCR4-NOT complexes, with its m6A-binding affinity significantly enhanced by SUMOylation (19, 59). Additionally, YTHDF1 and YTHDF3, both cytoplasmic m6A readers, enhance the translation efficiency of target mRNAs and, in some instances, promote their degradation (19, 60, 61). As for nuclear m6A readers, YTHDC1 regulates mRNA fate through multiple mechanisms, such as mRNA splicing (17), nuclear body formation (62), and retrotransposon silencing (63). YTHDC2,localized in both the nucleus and cytoplasm (64), modulates deconjugase activity and influences mRNA decay and translation during spermatogenesis (65–67). Apart from the YTH family, IGF2BPs constitute a distinct group of m6A readers, recognizing m6A through their KH structural domains (20). The IGF2BP proteins (IGF2BP1/2/3) share similar structures, and their binding affinities for various target RNAs may be governed by their KH3 and KH4 structural domains (68).

#### 2.1.2 m1A

The m1A modification, similar to m6A, entails the methylation of the first nitrogen on adenosine and is governed by specialized writers, erasers, and readers. This modification predominantly occurs in tRNAs and rRNAs, particularly within GC-rich RNA sequences, impacting ribosomal tertiary structure, RNA stability, and translation efficiency (**Figure 1**, 69–71). tRNA methyltransferases 6 and 61A (TRMT6/61A) form a complex that exerts MTC-like effects by catalyzing the addition of m1A to t-loop-like RNA structures (72). tRNA methyltransferases 10C and 61B (TRMT10C/61B) respectively catalyze the m1A modification at positions 9 and 58 in mitochondrial tRNAs (73). Moreover, TRMT61B has a similar recognition mechanism for rRNA and tRNA (74). Additionally, NML, also known as RRP8, localizes to the nucleus where it methylates m1A on 28S rRNAs (75). AlkB homologs 1, 3 and 7 act as erasers in charge of m1A demethylation (76–78). YTHDF1, YTHDF2, YTHDF3, and YTHDC1 act as m1A-modified readers to fulfill their biological roles (79).

#### 2.1.3 m5C

m5C is a methylation modification at the 5th carbon atom of cytosine, found in mRNA and lncRNA, and enriched in cytoplasmic and mitochondrial rRNAs and tRNAs (**Figure 1**, 80, 81). To date, NSUN2 and NSUN6 are the only known m5C methyltransferases within the NSUN family that facilitate mRNA methylation by incorporating m5C, functioning as transcriptional modifiers. NSUN2 regulates the nucleoplasmic transport and RNA-binding affinity of the mRNA export adapter protein ALYREF, which specifically recognizes m5C, thereby influencing mRNA export (81). NSUN6 predominantly targets the 3 untranslated regions (3 UTRs) on the hairpin-like structural loops conserved sequence motif CTCCA, potentially participating in the quality control of translation termination fidelity (82). Two mechanisms for m5C “erasure” have been identified: first, oxidation by the TET family on RNA to produce 5-hydroxymethylcytosine (hm5C); and second, the conversion of 5-formylcytosine (f5C) in mitochondrial tRNA by α-ketoglutarate and iron(II)-dependent dioxygenases ALKBH1 and ABH1 (83–85). BX-1 and the Aly-REF export factor (ALYREF) act as m5C readers, affecting the stability, translation, and transcription of the RNAs they target (81, 86).

#### 2.1.4 m7G

The m7G modification involves the methylation of the 7th nitrogen atom in guanosine and primarily occurs at internal sites of rRNAs, tRNAs, miRNAs, as well as the 5 cap of mRNAs (**Figure 1**, 87, 88). Although no confirmed erasers or readers have been introduced for m7G yet, the m7G cap can undergo hypermethylation by trimethylguanosine synthase 1 (TGS1) to produce m2,2,7G or may be recognized by the eukaryotic translation initiation factor eIF4E, subsequently affecting RNA maturation, nuclear export, and translation (89, 90).

#### 2.1.5 mcm5s2U

The 5-methoxycarbonylmethyl-2-thiourea modification (mcm⁵s²U), initiated by cm⁵U and mcm⁵U modifications on wobble uridines, is facilitated by human tRNA methyltransferase 9-like protein (TRM9L) and AlkB homologue 8 (ALKBH8) (**Figure 1**, 91). Wobble uridines, located at the first nucleotide position of the anticodon stem loop in tRNA, are essential for accurate mRNA translation and efficient protein synthesis (92, 93).

#### 2.1.6 A-to-I modification

The A-to-I modification involves the selective hydrolytic deamination of adenosine to inosine (A-to-I editing), a process primarily regulated by the family of double-stranded RNA-specific adenosine deaminases, notably ADAR1, ADAR2, and ADAR3 (**Figure 1**, 94). ADAR1 and ADAR2 both mediate A-to-I editing in cellular RNA (95).

#### 2.1.7 Pseudouridylation Ψ

The C5-glycosidic isoform of uridine, pseudouridine Ψ, is the most prevalent RNA modification, primarily found in tRNAs and rRNAs (**Figure 1**, 70, 96). Pseudouridylation occurs via two distinct pathways: RNA-independent pseudouridylation, catalyzed by pseudouridine synthases (PUSs) without a template strand, and RNA-dependent pseudouridylation, which requires the box H/ACA small nuclear ribonucleoprotein RNA-protein complex (82, 97).

#### 2.1.8 Crosstalk between different RNA modification

Multiple RNA modifications are not isolated; rather, they are often interlinked, collaboratively regulating physiological and pathological states in the body. The vast majority of RNA modifications share similar regulatory mechanism, especially writers, allowing different modifications to be controlled by the same class of writers (**Table 1**), thus forming an interconnected regulatory network. Writers regulating m6A, m1A, and A-to-I modifications are not independent but exhibit significant cross-linking, which is closely associated with colorectal carcinogenesis, the tumor microenvironment (TME), drug sensitivity, and immunotherapy (98). Multiple RNA modifications can act on the same signaling pathway or target RNA to exert either synergistic or antagonistic effects. For example, m6A and m5C both modify FOXC2 mRNA, promoting gastric cancer cell growth (99, 100). Both m6A and A-to-I editing can alter c-MYC mRNA, contributing to the progression of hepatocellular carcinoma (101, 102). Similarly, m6A and Ψ modifications within the RAS pathway have been recognized for their oncogenic impact in colorectal cancer (103, 104). In pancreatic cancer, m6A stimulates the PI3K/Akt/mTOR pathway, driving cancer cell proliferation (105, 106), whereas m1A and m5C are linked with activation of the mTOR pathway and unfavorable prognoses (107, 108). Additionally, interactions between different RNA modifications have been observed. In breast cancer, METTL3-mediated m6A modification is regulated by ADAR1, which subsequently promotes breast cancer progression (109). Interactions among m6A, m5C, m1A, and m7G are also vital for TME regulation, immune infiltration, and immunotherapy in soft tissue sarcoma (STS) (110). The human body is a complex system, where crosstalk among multiple RNA modifications plays an essential role in disease development. Expanding research on RNA modification interactions holds significant clinical promise.

**Table 1:** The various “writers”, “readers” and “erasers” associated with RNA modifications.

Key protein	m6A	m1A	m5C	m7G	mcm5s2U	A to I	Ψ
Writers	METTL3 METTL14 METTL16 WTAP VIRMA RBM15 HAKAI ZC3H13	TRMT6 TRMT61A TRMT10C TRMT61B RRP8	NSUN2 NSUN6	METTL1/WDR4	TRM9L ALKBH8	ADAR1 ADAR2 ADAR3	PUSs
Erasers	FTO ALKBH5	ALKBH1 ALKBH3 ALKBH7	TETs ALKBH1 ABH1	unknown	unknown	unknown	unknown
Readers	YTHs HNRNPs IGF2BPs	YTHDF1 YTHDF2 YTHDF3 YTHDC1	YBX-1 ALYREF	unknown	unknown	unknown	unknown

### 2.2 RNA modification with hormone receptors

Sex hormones in the human body, primarily estrogen, progesterone, and androgens, are regulated by gonadotropins, including gonadotropin-releasing hormone (GnRH), luteinizing hormone (LH), and follicle-stimulating hormone (FSH). Additionally, prolactin (PRL) can reflect the secretion levels of sex hormones in the body. RNA modifications specifically regulate the synthesis, secretion, and ligand-receptor interactions of sex hormones in organisms, thereby influencing physiological and pathological processes.

As the most common m6A methyltransferase, the specific knockdown of METTL3 can alter various biological processes, with diverse and sometimes opposing effects in different cells and molecules. In testicular mesenchymal cells, ambient PM2.5 promotes METTL3-induced m6A modification of SIRT1 mRNA, leading to aberrant cellular autophagy, which inhibits testosterone synthesis and results in impaired spermatogenesis and infertility (111). Knockdown of METTL3 has been observed to significantly promote autophagic flow and increase testosterone production in testicular mesenchymal cells (111). Specific knockdown of the METTL3 gene in the endometrium stabilizes several mRNAs of estrogen-responsive genes, such as *Elf3* and *Celsr2*, while significantly reducing the expression levels of the progesterone receptor (PR) and its target gene *Myc* (112). Multiple m6A regulatory proteins can act synergistically to regulate hormone levels.

METTL3 suppresses the expression of androgen receptors in cardiac fibroblasts by introducing m6A modifications to AR mRNA, which are subsequently recognized by YTHDF2, leading to the degradation of AR-associated mRNA. This m6A modification by METTL3 enhances the binding of YTHDF2 at the modified sites, thereby reducing AR expression. This reduction rescues the inhibitory effects exerted by AR on glycolysis and cardiomyocyte proliferation, ultimately facilitating myocardial fibrogenesis (113). Researchers also found that applying antisense oligonucleotides (ASOs) to target METTL3 can restore Enzalutamide(an effective AR inhibitor)resistance *in vitro* and *in vivo* (114). In endometrial cells, METTL3-mediated m6A modification directly influences the mRNA of PR. Specifically, m6A modification in the 5′ untranslated region (5′-UTR) of PR mRNA enhances the translational efficiency of PR proteins in a YTHDF1-dependent manner, a process that is conserved between mice and humans (112). M6A-related proteins such as METTL3 and METTL14 have been reported to increase follicle-stimulating hormone (FSH) levels while decreasing luteinizing hormone (LH) and testosterone (T) levels in PCOS rats, thereby reducing apoptosis and autophagy in ovarian tissue and improving ovarian morphology (115). Additionally, hormones can regulate biological processes by influencing m6A modifications. For instance, FSH can enhance the transcriptional activity of the METTL3 promoter in osteoclasts by inducing the phosphorylation of cyclic AMP response element-binding protein (CREB), which increases the m6A methylation of cathepsin K (CTSK). This methylation enhances the stability of CTSK and promotes osteoclast migration (116).

Other RNA modifications are also associated with hormone receptors, including A-to-I editing and m5C modification. In prostate cancer cells, numerous nucleotide transitions within AR gene transcripts have been identified as mutations that coincide with potential A-to-I, U-to-C, C-to-U, and G-to-A RNA editing sites (117). Furthermore, NSUN2 stabilizes AR mRNA through m5C modification, creating a positive feedback loop that promotes prostate carcinogenesis (118). Research has shown that inhibiting AR leads to the rearrangement of the alternative polyadenylation (APA) subcomplex and disrupts the interaction between the cleavage stimulation factor (CSTF) complex and the cleavage and polyadenylation specificity factor (CPSF) complex (119). In breast cancer, the luminal androgen receptor influences APA subtypes in patients with triple-negative breast cancer (120). In estrogen receptor-positive (ER^+^) breast cancer, estradiol (E2), a potent proliferative agent, induces APA and 3′-UTR shortening, subsequently activating proto-oncogenes (121).

## 3 RNA modification in hormone-dependent cancer

### 3.1 Tumor microenvironment in hormone-dependent cancer

**Figure 2:**
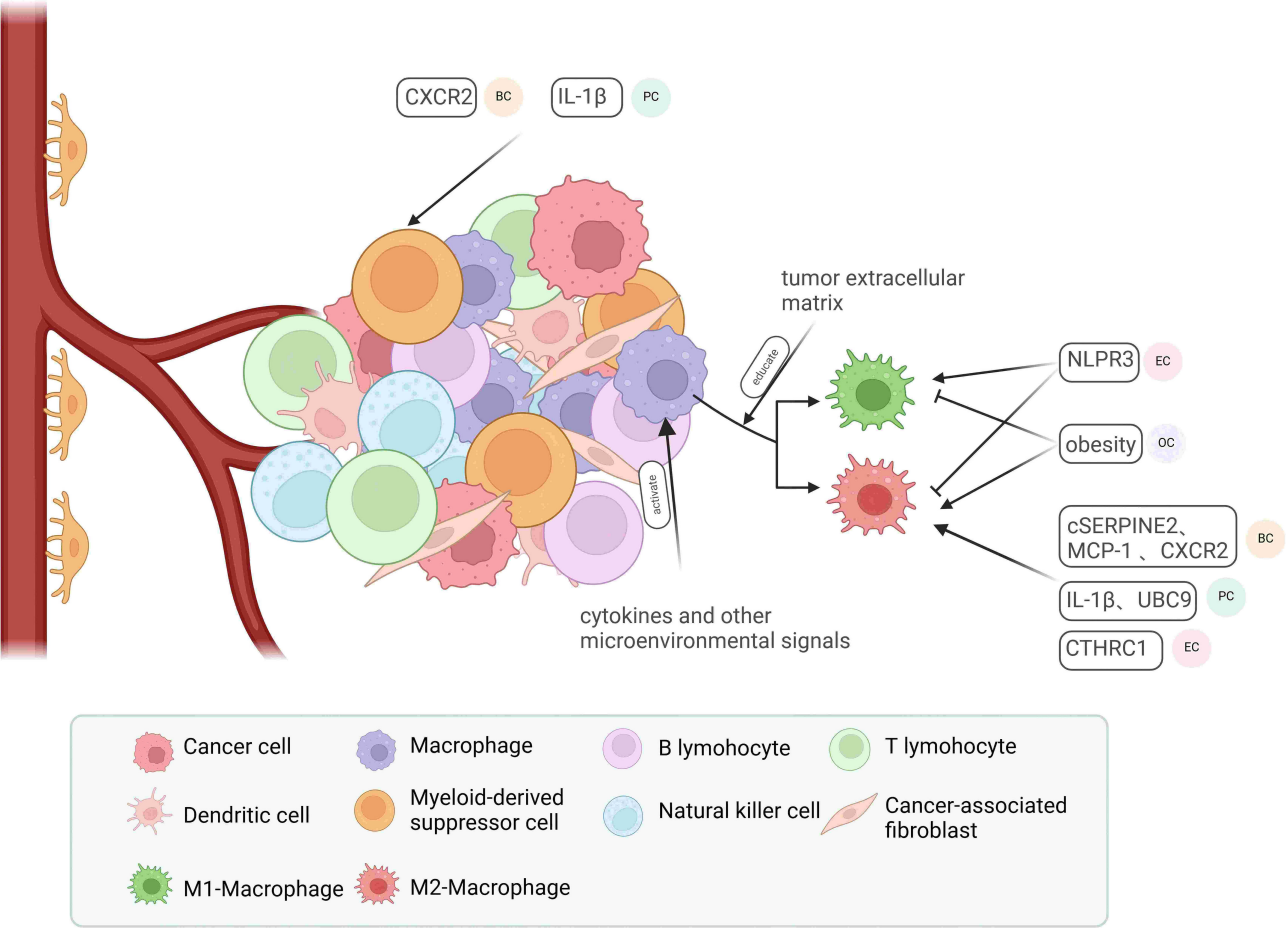
Major components of the tumor microenvironment and their regulatory factors. Macrophages in the tumor microenvironment can be activated by cytokines and various microenvironmental factors, leading to their polarization into M1-type or M2-type macrophages, influenced by multifaceted factors involving the tumor extracellular matrix. In endometrial cancer (EC), NLRP3 promotes M1-type macrophage polarization while inhibiting M2-type polarization. In ovarian cancer (OC), obesity exerts the opposite effect. In breast cancer (BC), pancreatic cancer (PC), and EC, cSERPINE2, MCP-1, CXCR2, UBC9, and CTHRC1 contribute to the recruitment of M2-type macrophages. Additionally, CXCR2 in BC and IL-1β in EC play roles in recruiting myeloid-derived suppressor cells (MDSCs).

Research indicates that the tumor microenvironment (TME), which consists of infiltrating immune cells such as tumor-associated macrophages (TAMs) and myeloid-derived suppressor cells (MDSCs), along with stromal cells like cancer-associated fibroblasts (CAFs) and endothelial cells (122), plays a pivotal role in tumor development. It fosters tumor progression through complex interactions between cells and the extracellular matrix (ECM) (**Figure 2**) (123, 124).

Tumor-associated macrophages (TAMs) are the most prevalent immune cells in the tumor microenvironment, crucially supporting tumor progression and immune modulation (125). With their phagocytic and cytotoxic capabilities, macrophages are recognized as immunoreactive cells that can polarize into anti-tumor M1 macrophages or pro-tumor M2 macrophages in response to microenvironmental signals. TAMs closely resemble M2 macrophages and are associated with the Th2 immune response, characterized by high levels of IL-10 and TGF-β production, and they secrete pro-tumorigenic cytokines that promote tumor progression (126–128). Furthermore, TAMs influence angiogenesis and enhance cell proliferation and metastasis by inhibiting CD8^+^ T cell activity (129, 130). Consequently, various factors can affect tumor development and metastasis by modifying the polarization and recruitment of TAMs (131). In EC, NLRP3 deficiency leads to macrophage polarization into pro-inflammatory M2-type macrophages (132). The tumor exosome cSERPINE2 (133), the chemokines MCP-1 (134) and IL-1β (135), and the secreted protein CTHRC1 (136) facilitate the progression of BC, EC, and pancreatic cancer (PC) by recruiting TAMs. An *in vitro* study demonstrated that the tumor extracellular matrix (ECM) can directly regulate macrophage populations in ovarian cancer tissues (137). MDSCs are also critical immunosuppressive components in the tumor microenvironment. Two main classes of MDSCs, granulocytic/polymorphonuclear MDSCs (PMN-MDSCs) and monocytic MDSCs (M-MDSCs), can be identified in humans and mice based on their origin, and both significantly suppress immune responses following prolonged exposure to cytokines released during chronic infections, inflammation, autoimmune diseases, and cancer (138). For instance, chronic psychological stress can recruit splenic MDSCs via CXCR2, promoting the formation of a metastatic pre-metastatic niche (PMN) in BC (139). Due to their significant immunosuppressive properties, TAMs and MDSCs are frequently studied as potential targets for tumor therapy. Approaches such as gene knockdown (140), blockade of key molecules (141–143), and remodeling of drug structure (144) aim to inhibit TAMs and MDSCs to achieve clinical benefits.

In most cancers, stromal cells are major components of the TME, playing critical roles in tumor metabolism, growth, and metastasis (145). Cancer-associated fibroblasts (CAFs), key constituents of the stroma, can be activated by various tumor-derived factors (146). CAFs exhibit enhanced expression of several markers, including α-smooth muscle actin (α-SMA), fibroblast activation protein (FAP), fibroblast-specific protein 1 (FSP1), platelet-derived growth factor receptor (PDGFR)-α/β, and poikilodulin (147), and the vast majority display pro-cancer effects (148). CAFs are highly heterogeneous, comprising multiple influential subgroups. In breast cancer, CD26^+^ and CD26-normal fibroblast populations are transformed into inflammatory CAFs (iCAFs) and myofibroblast CAFs (myCAFs), respectively (149). CD26^+^ normal fibroblasts (NFs) are converted into pro-tumorigenic iCAFs, which recruit myeloid cells via a CXCL12-dependent mechanism and promote tumor cell invasion through matrix metalloproteinase (MMP) activity (149). MyCAFs, located close to the tumor, are a subtype of CAFs. The molecular and functional diversity of myCAFs arises from diverse sources and activation mechanisms, among which TSPAN8^+^SIRT6^low^ myCAFs are linked to unfavorable outcomes in breast cancer patients (150). Similarly, in prostate cancer, androgen deprivation therapy (ADT) induces SPP1^+^ myCAFs, which are critical stromal components driving the progression of castration-resistant prostate cancer (CRPC) (151). Other non-classical subgroups include CD146+ CAFs, which promote endometrial cancer progression by inducing angiogenesis and vasculogenic mimicry (152), and αSMA^+^VIM^+^PDGFRβ^+^CAFs, which are correlated with lower tumor immune infiltration and shorter survival in ovarian cancer patients (153).

Additionally, recent studies have found that solid tumors are hypoxic and acidic, with the physiochemical aspects of the TME sustained by chaotic tumor perfusion, resulting in tumor progression and resistance to immunotherapy (154). Apart from recruiting immunosuppressive cells like MDSCs, tumor cells can evade the immune system in various ways. For instance, they modulate T cell responses by altering the levels of immune checkpoint molecules, particularly through the upregulation of PD-L1 (155). Moreover, tumor cells evade recognition and destruction by cytotoxic T cells by reducing MHC-I expression and impairing antigen presentation (156). They may also inhibit the production of CXCL9 and CXCL10, obstructing the infiltration of CXCR3+ effector cells into the tumor, thus facilitating immune evasion and limiting T cell infiltration (157).

### 3.2 RNA modification in immune system of hormone-dependent cancers

A growing number of studies have demonstrated that human malignancies are correlated with epigenetic alterations in RNA (158, 159). Previous studies have identified RNA modifications, particularly m6A, as playing a pivotal role in hormone-dependent cancers (160–163). These modifications are essential in regulating tumor growth and metastasis (33, 164).In BC, m7G has been linked to immune cell infiltration, including initial B cells, CD4^+^ memory resting and activated T cells, CD8^+^ T cells, regulatory T cells, resting and activated natural killer (NK) cells, M1 macrophages, and resting mast cells, with NCBP1 mRNA identified as the most prominent target of m7G (165).The related regulatory enzyme, RBM15B, along with its associated genes TCP1 and ANKRD36, and the RNA demethylase ALKBH family, particularly ALKBH7, are also associated with immune infiltration in breast cancer and are positively correlated with tumor development (166, 167). Furthermore, both m6A and m5C can disrupt DNA replication and affect the tumor immune microenvironment in PC (168, 169). As more relevant studies emerge, the understanding of how RNA modifications govern the immune system in hormone-dependent cancers has been progressively refined at the cellular and molecular levels (**Table 2**).

**Table 2:** The molecules, cells and mechanisms associated with the immunomodulatory role of RNA modifications in hormone-dependent cancers.

RNA modification	Related molecule	Immune cell	Key mechanism	Tumor	References
m6A	circATAD2 IGF2BP3	T cell	Enhancing PD-L1 mRNA stability and expression	BC	(170)
	METTL3 IGF2BP3	T cell	Upregulating PD-L1 expression and promoting stabilization of PD-L1 mRNA	BC	(171)
	YTHDF1	T cell	Enhancing PD-L1 transcriptional stability	PC	(172)
	m6A RNA demethylase inhibitor meclofenamic acid	T cell	Decreasing the stability of PD-L1 transcripts	PC	(173)
	circNFIX IGF2BP1/2/3	T cell	Activating IL-6R/JAK/STAT3 signaling and enhancing PD-L1 expression	OC	(174)
	HNRNPC	Treg cell CD8^+^T cell	Characteristic m6A gene profiles were associated with immune responses, in which HNRNPC as a marker protein enhances treg cell activation and suppresses effector CD8^+^T cells.	PC	(175)
		CD4^+^T cell Macrophage Mast cell	The levels of macrophages, mast cells and CD4^+^T cells were significantly correlated with m6A-related genes.	PC	(176)
	KIAA1429	B cell	Inhibition of memory B cell infiltration	BC	(177)
		B cell Dendritic cell	Elevated m6A levels were accompanied by elevated dendritic cell and B cell levels.	EC	(178)
	ALKBHs (especially ALKBH8)	All tumor- related immune cells	Associated with immune infiltration and promotes tumor development	BC	(167)
m7G	NCBP1 mRNA	All Tumor -related Immune cells	Associated with low immune status and poorer prognosis	BC	(165)
	METTL1	CD8^+^T cell Macrophage	The depletion of METTL1 promotes the biogenesis of 5’tRNA-derived small RNA,which correlates with increased pro-inflammatory immune cell polarization and CD8^+^T cell inflation.	PC	(179)
m5C	TETs,NUSNs,etc	CD8^+^T cell Macrophage B cell	m5C regulatory genes were associated with immune cell levels and tumor prognosis.	PC	(180)
A to I	ADAR1 DDX3X	CD8^+^T cell Dendritic cell	Activation of the cytoplasmic dsRNA pathway increases tumor infiltration by CD8^+^T cell and DC.	BC	(181)
RNA modification	RBM15B	All tumor- related immune cells	Associated with low immune status and poorer prognosis.	BC	(166)
	ALYREF ZC3H13 WTAP METTL1	CD4^+^T cell Macrophage B cell	The risk scores based on the four-DERRG signature showed a positive correlation with CD4^+^ memory resting T cells, while demonstrating a negative correlation with M1 macrophages and plasma cells	OC	(182)
	Related writers	All tumor- related immune cells	Correlated with high expression of tumor infiltration-associated cells and B-cell receptor signaling pathways.	OC	(183)

#### 3.2.1 T cells

RNA modification promotes tumor immune escape by regulating immune checkpoint molecule expression on T cells. Researchers identified a 4-DERRG signature based on 59 RNA modification-associated regulatory genes (ALYREF, ZC3H13, WTAP, and METTL1) and accordingly categorized the OC patients into two distinct groups, showing significant differences in the immune checkpoint molecule CD276. The regulation of immune checkpoint molecules by m6A is primarily mediated through PD-L1. METTL3-mediated m6A modification occurs in the 3-UTR of PD-L1 mRNA, and circATAD2 can bind to it, enhancing the level of m6A modification (170, 171).The m6A reader IGF2BP3 recognizes this modification, thereby increasing PD-L1 mRNA stability and expression (171). In OC, IGF2BP1/2/3 also recognize m6A modifications, positively regulating circNFIX expression, which activates downstream JAK/STAT3 signaling and enhances PD-L1 expression (174).Thus, m6A-modified PD-L1 may serve as a potential therapeutic target. In BC, ADAR1 synergizes with DEAD-box RNA helicase 3X (DDX3X) to activate the cytoplasmic dsRNA pathway, increasing tumor infiltration of CD8^+^ T cells and DC cells (181). In PC, the reader YTHDF1 promotes the progression by regulating androgen function-related gene TRIM68 (172). For enhanced photothermal immunotherapy of PC, cyclodextrin-functionalized gold nanorods can deliver the m6A RNA demethylase inhibitor meclofenamic acid, thereby enhancing m6A methylation of mRNAs and decreasing the stability of PD-L1 transcripts (173). Besides, investigators have discovered that the m7G transferase METTL1 is highly expressed in both primary and advanced prostate tumors. Simultaneously, upon METTL1 deletion, the absence of m7G tRNA methylation promotes the generation of a new class of non-coding small RNAs originating from 5 tRNA fragments (179). These small RNAs regulate translation and support the production of key regulators essential for antitumor immune responses (179). These regulators are crucial for promoting CD8^+^ T cell infiltration and enhancing antitumor effects (179). Similarly, after clustering according to the regulatory genes of m5C (TET1, TET3, DNMT3B, YBX1, NSUN2, NSUN6, NOP2) in patients with PC, significant differences in CD8^+^ T cell infiltration were observed between the two clusters, with a strong negative correlation to patient prognosis (180).

#### 3.2.2 Macrophages

RNA modification regulates macrophages mainly by altering the number or proportion of M1 and M2 type macrophages. For instance, circITGB6 specifically interacts with the KH1–2 domain of IGF2BP2, leading to increased mRNA stability of FGF9, leading to increased mRNA stability of FGF9 (184). This interaction further encourages the polarization of TAM towards the M2 phenotype, thereby inducing cisplatin resistance in OC (184). In PC, the removal of METTL1 results in the downregulation of anti-inflammatory cytokines, such as macrophage colony-stimulating factor (M-CSF), IL-10, and IL-13, which also promote M2 macrophage polarization (179, 185). In OC and BC, ALKBH3 enhances the half-life of CSF-1 mRNA by removing m1A from the GC-rich region of the 5 UTR of CSF-1 mRNA, facilitating macrophage recruitment and tumor invasion (186).

#### 3.2.3 Other cells

In OC, based on the six lncRNA subgroups of RNA modification-associated writers (m6A, m1A, APA, and A-I), tumor-infiltrating cells such as mast cells, neutrophils, and B-cell receptor signaling pathways were highly expressed in the high-risk group (14). Moreover, m6A writer KIAA1429 was positively correlated with various advanced tumors such as BC, and negatively correlated with memory B-cell infiltration (177). In addition, in EC clusters classified by hypoxia genes, elevated m6A levels were observed alongside increased infiltration of B cells and dendritic cells (DCs) in the high-risk group (178).

### 3.3 Targeting regulators of RNA modification to treat hormone-dependent cancer

Despite being a relatively new field, drugs targeting RNA modifications are gradually transitioning from the laboratory to the public eye. However, fewer studies have been specifically conducted on the four hormone-dependent cancers, and the following is only a list of drugs of general interest that can potentially be used on hormone-dependent cancers. For m6A, Several inhibitors targeting FTO and ALKBH5 have been developed to impede the progression of various cancers, such as R-2-hydroxyglutarate (R-2HG), FB23-2, IOX1, IOX3, Rhein, Entacapone, and meclofenamic acid (80, 97). Regarding m5C, NSUN2 is upregulated in both BC and PC, and its expression can be reduced by inhibiting sphingosine kinase (SPHK), which maintains sphingolipid balance during cell growth (187–189). Consequently, the SPHK1 inhibitor SK1 emerges as a potential agent for cancer treatment by targeting NSUN2 expression (187–189). Moreover, research on pseudouridine identifies pyrazoline and 5-fluorouracil as common DKC1 inhibitors, employed clinically as anticancer agents (97, 190). In the context of A-to-I editing, 8-azaadenosine and 8-chloroadenosine function as ADAR1 inhibitors, but their limited specificity temporarily precludes clinical application (191, 192).

Furthermore, while no clinical trials have been conducted to date, emerging mechanistic studies suggest that specific RNA modifications may exert dual therapeutic effects in hormone-dependent tumors: either enhancing treatment efficacy or paradoxically promoting drug resistance. In OC, RNA modifications can remodel the tumor microenvironment by upregulating immunogenic RNAs, thereby reversing tumor immune evasion phenotypes and potentially restoring clinical responsiveness to immunotherapy in previously non-responding patients (193). For instance, Y-box binding protein 1 (YBX1) has been shown to enhance homologous recombination proficiency and resistance to platinum-induced stress in OC through m5C modification (194). In breast cancer (BC), METTL3 knockdown significantly increases chemosensitivity to doxorubicin via modulation of the EGF/RAD51 signaling axis (195). Intriguingly, METTL3 depletion has also been found to activate the CDKN1A/EMT pathway and m6A-BAX/caspase-9/-3/-8 cascade, thereby promoting proliferation, migration, and drug resistance in hormone receptor-positive HER2-negative breast cancer (HR+HER2-BC) (196). These findings underscore the complex regulatory networks of RNA modifications in cancer therapeutics, necessitating comprehensive mechanistic elucidation and systematic clinical validation to delineate their therapeutic potential versus risk profiles.

## 4 Conclusion and perspectives

This review underscores the crucial role of RNA modifications in regulating the progression and immune landscape of hormone-dependent cancers, including breast, ovarian, endometrial, and prostate malignancies. These modifications facilitate tumor growth and metastasis by modulating key immunoregulatory pathways, such as PD-L1 expression, immune cell infiltration, and cytokine signaling, revealing their potential to improve cancer diagnosis and therapy.

Despite increasing recognition of RNA modifications in cancer, the precise molecular mechanisms—especially how these modifications integrate with hormone receptor signaling and shape the immune microenvironment—remain only partially understood. Future studies should elucidate the specific pathways by which RNA modifications influence immune regulation and hormone receptor activity. As RNA modifications affect both hormone receptor function and immunogenic pathways (e.g., PD-L1), there is a compelling rationale for combining hormone therapies (e.g., anti-estrogen, anti-androgen) with immunotherapies or RNA modification inhibitors. Such combination strategies may enhance tumor susceptibility to immune-mediated destruction and mitigate therapeutic resistance.

Although therapeutic applications remain challenging, mounting evidence highlights the significant role of RNA modifications in orchestrating immune regulation and driving hormone-dependent tumor progression. Further investigation into the detailed mechanisms underlying these modifications holds promise for developing more effective and precisely targeted interventions against hormone-dependent cancers.
